# Hydrothermal Synthesis and Gas Sensing of Monoclinic MoO_3_ Nanosheets

**DOI:** 10.3390/nano10050891

**Published:** 2020-05-07

**Authors:** Teodóra Nagyné-Kovács, Levente Studnicka, István Endre Lukács, Krisztina László, Pawel Pasierb, Imre Miklós Szilágyi, György Pokol

**Affiliations:** 1 Department of Inorganic and Analytical Chemistry, Budapest University of Technology and Economics, Műegyetem rakpart 3, H-1111 Budapest, Hungary; kovacs.teodora@mail.bme.hu (T.N.-K.); studnickalevi@gmail.com (L.S.); pokol.gyorgy@ttk.mta.hu (G.P.); 2 Research Institute for Technical Physics and Materials Science, Eötvös Loránd Research Network, Konkoly Thege M. út 29-33, H-1121 Budapest, Hungary; lukacs.istvan@energia.mta.hu; 3 Department of Physical Chemistry and Materials Science, Budapest University of Technology and Economics, Műegyetem rakpart 3, H-1111 Budapest, Hungary; klaszlo@mail.bme.hu; 4 Faculty of Materials Science and Ceramics, AGH University of Science and Technology, Al. Mickiewicza 30, 30-059 Kraków, Poland; ppasierb@agh.edu.pl; 5 Research Centre for Natural Sciences, Eötvös Loránd Research Network, Magyar tudósok körútja 2, H-1117 Budapest, Hungary

**Keywords:** hydrothermal, monoclinic MoO_3_, nanosheet, CrCl_3_, gas sensing

## Abstract

Effects of different reaction parameters in the hydrothermal synthesis of molybdenum oxides (MoO_3_) were investigated and monoclinic (β-) MoO_3_ was prepared hydrothermally for the first time. Various temperatures (90/210 °C, and as a novelty 240 °C) and durations (3/6 h) were used. At 240 °C, cetyltrimethylammonium bromide (CTAB) and CrCl_3_ additives were also tested. Both the reaction temperatures and durations played a significant role in the formation of the products. At 90 °C, h-MoO_3_ was obtained, while at 240 °C the orthorhombic (α-) MoO_3_ formed with hexagonal rod-like and nanofibrous morphology, respectively. The phase transformation between these two phases was observed at 210 °C. At this temperature, the 3 h reaction time resulted in the mixture of h- and α-MoO_3_, but 6 h led to pure α-MoO_3_. With CTAB the product was bare o-MoO_3_, however, when CrCl_3_ was applied, pure metastable m-MoO_3_ formed with the well-crystallized nanosheet morphology. The gas sensing of the MoO_3_ polymorphs was tested to H_2_, which was the first such gas sensing study in the case of m-WO_3_. Monoclinic MoO_3_ was found to be more sensitive in H_2_ sensing than o-MoO_3_. This initial gas sensing study indicates that m-MoO_3_ has promising gas sensing properties and this MoO_3_ polymorph is promising to be studied in detail in the future.

## 1. Introduction

Molybdenum oxides (MoO_3_) are considerable materials among molybdenum (Mo) compounds due to their excellent physical and chemical properties. MoO_3_ is one of the most important starting materials for other Mo compounds, such as for sodium molybdate, ammonium di- or heptamolybdate, or the Mo metal itself. MoO_3_ is also used as a catalyst in the industrial production of acrylonitrile from propylene and ammonia. However, the constant interest in nanomaterials has made also nano MoO_3_ attractive, which has been proven to be a promising candidate in many fields, e.g., (photo)catalysis, gas sensing, electrochemical cells, or even in forensic science and photothermal therapy [1,2,3,4,5,6,7,8,9,10,11,12,13].

MoO_3_ has four allotropes, each composed of differently linked MoO_6_ octahedra, the position of which determines phase stability. The most commonly studied MoO_3_ phases are the thermodynamically stable orthorhombic (o-) or α-MoO_3_ and metastable monoclinic (m-) or β-MoO_3_. Both phases have excellent physical and chemical properties such as refractive index, band gap, and mechanical hardness. Further crystalline phases are the metastable high-pressure ε-MoO_3_ and the relatively more stable hexagonal (h-) MoO_3_ [14,15,16].

In the case of o-MoO_3_, octahedra are distorted and form bilayers, which build up the well-known stratified structure. These planar bilayers are composed of chains of MoO_6_ octahedra sharing corners and edges in the specified direction and are held together vertically by weak Van der Waals forces. In contrast, the crystal lattice of the metastable m-MoO_3_ is similar to the cubic ReO_3_, where not layered, but 3D crystal structure forms due to the corner-sharing of distorted octahedra in the three directions. The h-MoO_3_ phase is constructed by such chains in which octahedra are linked by their corners. These chains connect in such a way to form a hexagonal structure with the typical 1D hexagonal and trigonal channels. The hexagonal channels may contain small ions (e.g., alkali metal or ammonium ions) or water molecules, similar to hexagonal WO_3_ [14,15,16,17].

There are several reports about the hydrothermal preparation of MoO_3_, but mostly only o- and h-MoO_3_ are synthetized. Although the synthesis of the m-MoO_3_ phase has been carried out by many methods (spray pyrolysis, pulsed laser thin-layer deposition, molecular beam epitaxy, o-MoO_3_ conversion, solution-phase reaction, etc.), it has not been prepared hydrothermally yet in pure form, only as a part of a mixture of MoO_3_ polymorphs [18]. On the other hand, it would be useful because the hydrothermal method is simple, does not use high temperatures (usually it operates only between 100 and 250 °C), results in highly crystalline, monodisperse products, and metastable phases can be also synthetized by it. Based on the literature, the highest temperatures used in experiments to prepare MoO_3_ are typically 200–210 °C. However, it would be worth trying to use higher reaction temperatures as well, considering how important a small change in the reaction parameters can be during the hydrothermal synthesis in general, regarding the crystalline phase and morphology of the products. Consequently, neither the different reaction times nor the role of CTAB (cetyltrimethylammonium bromide) and CrCl_3_ additives have been studied yet at temperatures above 200–210 °C in the hydrothermal synthesis of MoO_3_. Furthermore, so far the gas sensing properties mostly of o- and h-MoO_3_ were tested [10,19,20,21,22,23,24,25,26,27,28,29,30]. To the best of our knowledge, there is only one paper reporting the gas sensing of m-MoO_3_, where very brief gas sensing data are given using H_2_O and CO_2_ as test gases [31].

In the present investigation, we studied different reaction parameters in the hydrothermal synthesis of MoO_3_, focusing on the changes in the obtained crystalline phases and morphologies. We investigated the effect of reaction temperature at 90, 210, and 240 °C (using as high as 240 °C for the first time), and duration applying 3 and 6 h reaction times. Finally, we tested CTAB and CrCl_3_ additives for the first time at 240 °C. We examined the crystalline phases and morphologies by X-ray diffraction (XRD) and scanning electron microscopy (SEM), respectively. The as-prepared different MoO_3_ allotropes were further studied by energy dispersive X-ray spectroscopy (EDX) and specific surface area measurements, moreover by Fourier transformed infrared (FT-IR), Raman, and ultraviolet-visible (UV–Vis) spectroscopies. Their band gap energies were also determined and their gas sensing properties were analyzed towards H_2_.

## 2. Materials and Methods

### 2.1. Materials and Equipment

Ammonium heptamolybdate tetrahydrate (≥99.0%), hexadecyltrimethylammonium bromide (≥96.0%), and chromium(III) chloride hexahydrate (≥98.0%) were purchased from Sigma-Aldrich (Budapest, Hungary) and used as received. Concentrated HNO_3_ (65%) was ordered from Molar Chemicals (Halásztelek, Hungary), while ethanol (96 *V/V*%) from WVR (Budapest, Hungary). Ion exchanged water was used throughout the experiments.

For the hydrothermal reactions a 45 mL stainless steel, Teflon-lined autoclave (4744, General Purpose Vessel, Parr Instruments, Moline, IL, USA), and a Nabertherm (Lilienthal, Germany) muffle-furnace (L9/11/B410) were used.

### 2.2. Hydrothermal Reactions

For every synthesis, 0.5 g of ammonium heptamolibdate (AHM) was dissolved in 25 mL of ion exchanged water, and then 5 mL cc. HNO_3_ was added. To study the effect of various additives, 0.1 g of CTAB or CrCl_3_ was also dissolved in the selected reactions. After stirring the solution for a couple of minutes, it was transferred into the autoclave, which was put into the electric furnace at particular temperatures and for different durations. After the hydrothermal treatment, the precipitates were filtered and washed several times with water and ethanol. Finally, the samples were dried at 60 °C for 2 h.

The hydrothermal reactions and their conditions are listed in Table 1.

### 2.3. Characterization

X-ray powder diffraction (XRD) measurements were carried out by a X’Pert Pro MPD diffractometer (PANalytical, Almelo, Netherlands) with Cu Kα radiation (*λ* = 0.15418 nm). For studying the morphology of the samples, a LEO 1540 XB electron microscope (Zeiss, Oberkochen, Germany) was used, while energy-dispersive X-ray spectroscopy (EDX) analyses were performed using a JEOL JSM 5500-LV instrument (JEOL, Tokyo, Japan). FT-IR spectra were recorded by a Perkin Elmer 2000 FT-IR spectrometer (Perkin Elmer, Boston, MA, USA) between 450 and 4000 cm^−1^ using KBr pellets (1 mg sample/300 mg KBr). Raman spectra were taken by a Jobin Yvon LabRam spectrometer (Horiba, Kyoto, Japan) equipped with an Olympus BX41 optical microscope (Olympus, Tokyo, Japan) using a frequency-doubled Nd-YAG laser (532 nm), while for diffuse reflectance UV–Vis measurements, a Jasco V-570 UV/ VIS/ NIR spectrometer (Jasco, Tokyo, Japan) was used. The band gaps of the products were calculated based on the UV–Vis spectra using *αhυ = A (hυ − E_g_)^n^* equation, where *α*, *hυ*, *A*, and *E_g_* are equal to the molar absorption coefficient, photon energy, general constant, and band gap energy, respectively. The *n* depends on the type of the electron transition in the compound and is ½ (direct allowed) for MoO_3_. Using the Tauc-plot, which is plotting *(αhυ)2* against *hυ*, and then drawing a tangent line onto the linear range and extrapolating, the band gap energy can be determined. The Kubelka-Munk function was used to approximate the value of *A*. The N_2_ adsorption was measured at −196 °C with a Nova2000e (Quantachrome) computer-controlled apparatus and the apparent surface area (SBET) of the samples were calculated from the Brunauer–Emmett–Teller (BET) model [32].

### 2.4. Gas Sensing Tests

For the experiments, Al_2_O_3_ ceramic sensor plates with interdigital platinum electrodes were used. First, with the usage of gold paste gold strands were attached onto the electrodes. Then, the sensor plate was affixed to the end of a vertical ceramic tube, and it was connected to a circuit, to which an electrical resistance meter (HP 34410A) was connected. The samples were suspended in one drop of ethanol in an Eppendorf tube, and a small amount of the suspension was transferred to the electrode. For heating the sensor chips during the gas sensing measurements, an online, temperature, and atmosphere controlled furnace was utilized, which hosted the interdigital electrodes mounted on the vertical ceramic tube (see details in [33]). In the experiments, H_2_ gas with different concentrations (25,000/830/2500/5000/7500 and 10,000 ppm) was used and the carrier gas was N_2_. After the temperature was set, the gas mixture was introduced to the sample and the change in the electrical resistance of the sensor material was detected. The electrical resistance was measured for 1 h at each concentration.

## 3. Results and Discussion

### 3.1. Role of Reaction Temperature and Duration on the Formation of MoO_3_

**Figure 1:**
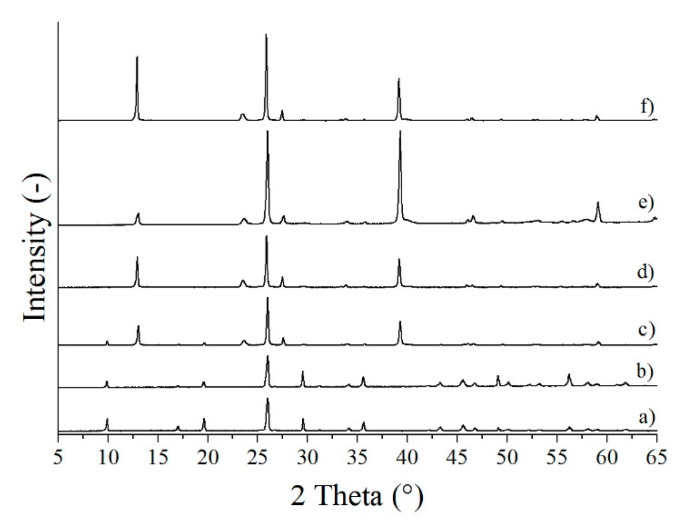
XRD patterns of the samples synthetized in the hydrothermal reaction of AHM and HNO_3_ at different temperatures and durations, (**a**) MoO_3_-1 (90 °C, 3 h), (**b**) MoO_3_-2 (90 °C, 6 h), (**c**) MoO_3_-3 (210 °C, 3 h), (**d**) MoO_3_-4 (210 °C, 6 h), (**e**) MoO_3_-5 (240 °C, 3 h), (**f**) MoO_3_-6 (90 °C, 3 h).

Based on their XRD patterns, MoO_3_-1 and MoO_3_-2 were identified as pure h-MoO_3_ (ICDD 065-0033, Figure 1a,b). Every reflection belonged to this phase indicating that no contaminations were present. MoO_3_-3, which were prepared at 210 °C for 3 h, was composed of the h- and o-MoO_3_ phase (ICDD 01-074-7909); however, the longer reaction duration, namely 6 h, resulted in pure o-MoO_3_ (Figure 1c,d). As the temperature was further increased to 240 °C, at both reaction times pure o-MoO_3_ was obtained (Figure 1e,f). All samples were crystalline, demonstrated by the sharp, intensive peaks.

**Figure 2:**
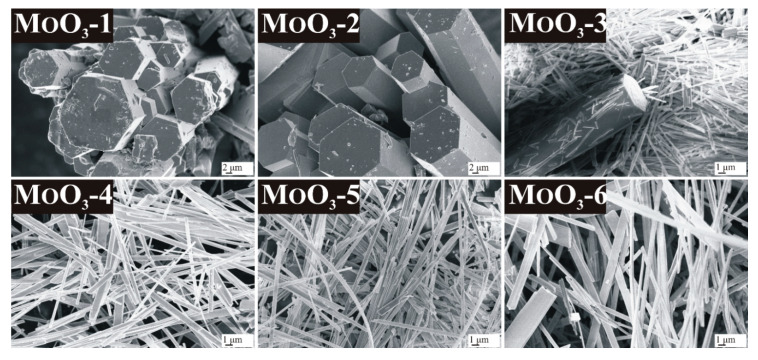
SEM images of the samples synthetized in the hydrothermal reaction of AHM and HNO_3_ at different temperatures and durations, MoO_3_-1 90 °C, 3 h, MoO_3_-2: 90 °C, 6 h, MoO_3_-3: 210 °C, 3 h, MoO_3_-4: 210 °C, 6 h, MoO_3_-5: 240 °C, 3 h, MoO_3_-6: 240 °C, 6 h.

According to SEM images, the h-MoO_3_ phases prepared at 90 °C were crystallized in a well-distinguished hexagonal form at both reaction durations (Figure 2, MoO_3_-1-2). The rods had a diameter of 7–12 μm when a 3 h duration was applied, and 8–18 μm diameter with a 6 h reaction time; however, their length was 10–50 μm in both cases. It can be also observed that the MoO_3_-2 sample had a much smoother surface and sharp edges due to the longer reaction time.

In the case of MoO_3_-3, the sample contained both h- and o-MoO_3_ phases, whose presence was obvious based on the observed morphologies. In addition to the robust hexagonal rods, 200–500 nm thick fibers formed, with longer (30–50 µm) lengths (in some cases they were broken into shorter, i.e., 1–2 µm pieces), referring to the o-MoO_3_ phase (Figure 2, MoO_3_-3). The morphology of MoO_3_-4 was, however, homogenous, and the sample was composed of 250–800 nm thick and 10–50 µm long fibers owing to the presence of only the o-MoO_3_ phase (Figure 2, MoO_3_-4).

When increasing the temperature to 240 °C, both reaction durations resulted in o-MoO_3_ with fibrous morphology. The diameter of the fibers was 250–800 nm when 3 h was applied, but the fibers became 500–1000 nm thick with 6 h reaction time. Their length was several 10–50 μm in both cases (Figure 2, MoO_3_-5-6).

Based on these results, it was found that the crystalline structure and morphology of the obtained products can be easily influenced by the appropriate choice of reaction temperature and duration. The lower temperature of 90 °C resulted in a pure hexagonal MoO_3_ structure with the characteristic hexagonal micrometer thick rod-like morphology, whereas the higher temperature, namely 240 °C led to a pure orthorhombic MoO_3_ phase, which was crystallized in the form of nanofibers. The transformation of the hexagonal to orthorhombic phase could be observed using 210 °C, and 3 h synthesis parameters, where both polymorphs were present with both types of morphologies. However, not only the reaction temperature but also the duration played an important role in this phase transformation, as it took place completely only when a long enough reaction time (6 h) was applied.

### 3.2. Role of Additives on the Formation of MoO_3_

**Figure 3:**
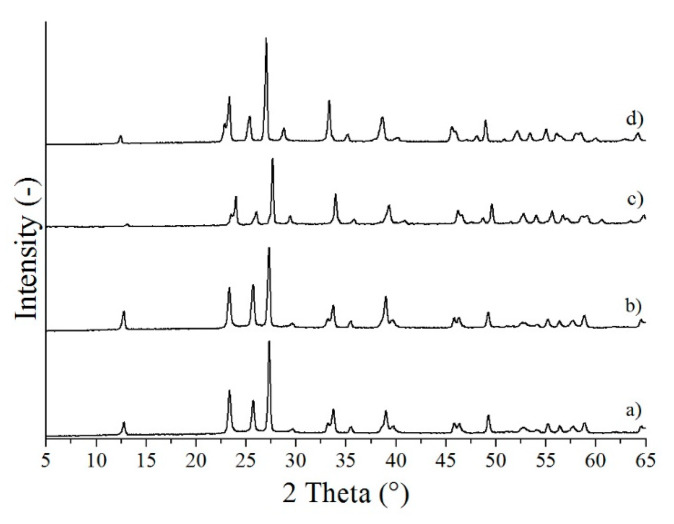
XRD patterns of the samples synthetized in the hydrothermal reaction of AHM and HNO_3_ with various additives, (**a**) MoO_3_-7 (CTAB, 240 °C, 3 h), (**b**) MoO_3_-8 (CTAB, 240 °C, 6 h), (**c**) MoO_3_-9 (CrCl_3_, 240 °C, 3 h), (**d**) MoO_3_-10 (CrCl_3_, 240 °C, 6 h).

When the CTAB additive was used, samples MoO_3_-7 and also MoO_3_-8 were identified as pure crystalline o-MoO_3_, similarly to those reactions, where no additive was used (Figure 3a,b). Experiments with the addition of CrCl_3_, however, resulted in a pure, metastable m-MoO_3_ phase (ICDD 01-085-2405) at both 3 and 6 h reaction durations, which is unprecedented in the literature (Figure 3c,d).

**Figure 4:**
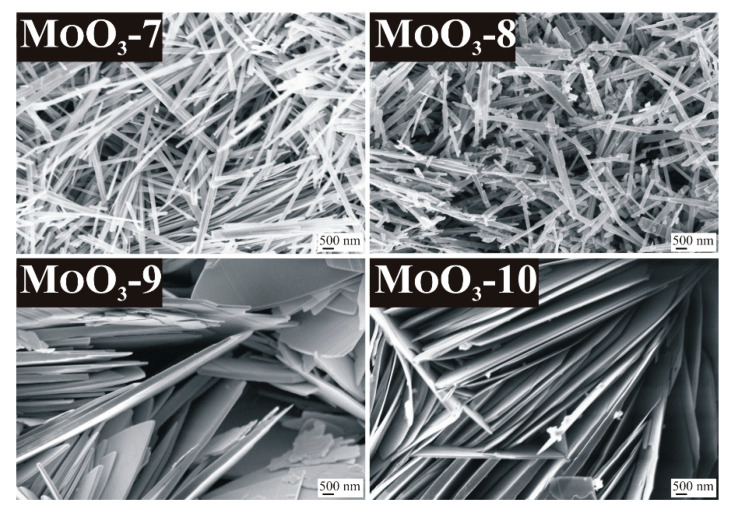
SEM images of the samples synthetized in the hydrothermal reaction of AHM and HNO_3_ with various additives, MoO_3_-7: CTAB, 240 °C, 3 h, MoO_3_-8: CTAB, 240 °C, 6 h, MoO_3_-9: CrCl_3_, 240 °C, 3 h, MoO_3_-10: CrCl_3_, 240 °C, 6.

In the case of CTAB additive, the uniform morphology typical of o-MoO_3_ formed here as well, containing fibers with a diameter of 200–500 nm and a length of 10–30 µm, which were slightly fragmented in the case of 6 h reaction time (Figure 4, MoO_3_-7-8).

The new metastable m-MoO_3_ was crystallized in the form of nanosheets, in contrast with the former, longitudinal shapes (columns, rods) of the other phases. The sheets had an average thickness of 100–250 nm when 3 h reaction time was applied and it increased to 100–500 nm at 6 h duration, due to the longer reaction time which favored crystal growth. The sheets were 10–20 μm long in both cases and were arranged almost parallel to each other (Figure 4, MoO_3_-9-10).

Examining the role of the additives, it was concluded that the CTAB additive did not influence the formation of crystalline phases during the reaction, as it also led to pure o-MoO_3_. On the other hand, CTAB affected the morphology resulting in a significant reduction in the diameter of the fibers. Without the additive the fiber diameter of o-MoO_3_ was 250–800 nm and 500–1000 nm, depending on the duration (3 h and 6 h, respectively), but with CTAB it became much thinner, 100–250 nm and 100–500 nm in the 3 and the 6 h experiments, respectively. In contrast, the addition of CrCl_3_ additive resulted in the formation of the metastable m-MoO_3_, which has not been prepared by hydrothermal synthesis previously yet. Its role in directing crystal growth also became obvious as it led to nanosheet morphology, presumably due to its favored adherence to certain crystal planes resulting in inhibition of crystal growth in specified directions.

### 3.3. Further Investigation of the Different MoO_3_ Phases

For a more detailed examination of hexagonal (MoO_3_-1), o- (MoO_3_-8), and m-MoO_3_ (MoO_3_-10) phases, various methods were used.

Results of the specific surface area measurement demonstrated significant differences among the phases, which can be explained by their completely different morphologies (Table 2 SBET). The smallest (0.21 m^2^/g) specific surface area belonged to the hexagonal MoO_3_ phase, which was crystallized in the form of the most robust hexagonal rods with a diameter and length of several µm. The orthorhombic phase had the largest (9.7 m^2^/g) surface area, which is attributed to its nanofibrous morphology, whereas the size of monoclinic MoO_3_ nanosheets surface was 2.9 m^2^/g.

Studying the elemental composition of the phases by EDX, it was found that the ratio of the main elements (Mo, O) was close to the stoichiometric Mo:O = 1:3 ratio, and there were no other elements referring to contaminations in the samples.

**Figure 5:**
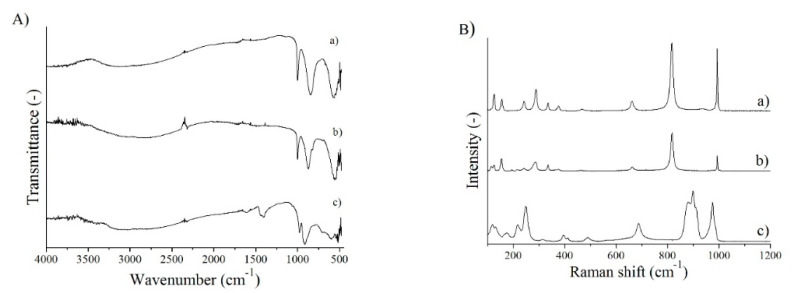

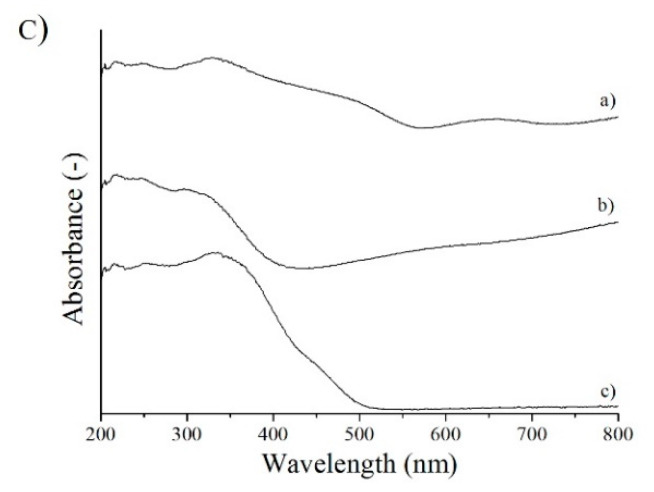
FT-IR (**A**), Raman (**B**), and UV–Vis (**C**) spectra of different MoO_3_ phases, (**a**) MoO_3_-10, monoclinic phase (CrCl_3_, 240 °C, 6 h), (**b**) MoO_3_-8, orthorhombic phase (CTAB, 240 °C, 6 h), (**c**) MoO_3_-1, hexagonal phase (90 °C, 3 h).

In the FT-IR spectra, the characteristic metal-oxygen (Mo-O) vibrations in the range below 1000 cm^−1^ can be observed, by the help of which the phases can be distinguished (Figure 5A). Since the orthorhombic and monoclinic crystal structures are very similar, their spectrum is hardly different, unlike the hexagonal phase, where the difference is clearly visible. The intensive peak around 1000–900 cm^−1^ refers to the Mo=O band, while the other one at 550–600 cm^−1^ belongs to the Mo-O valence vibration. In the case of o-MoO_3_, another less intensive peak appears at 830 cm^−1^, which is attributed to the Mo-O-Mo deformation vibration and it is not present in the spectrum of the monoclinic phase. In contrast, in the spectrum of the h-MoO_3_ phase, a band can be observed at 700–750 cm^−1^ referring to the Mo-O vibration, which does not appear in the case of the other two phases. In addition, peaks around 1400 and 3200 cm^−1^, moreover at 1600 and 3400 cm^−1^ belong to the N-H vibrations of the NH_4_^+^ group and to the O-H vibrations of O-H groups and water molecules, respectively. These bands are not detectable in the case of orthorhombic and monoclinic MoO_3_ phases. This phenomenon can be explained by the fact that hexagonal channels usually store small ions, e.g., NH_4_^+^ or molecules e.g., H_2_O which stabilize the structure, similar to h-WO_3_ [2,11,15,34,35].

In the Raman spectra, the distinctive bands of o-MoO_3_ at 990, 820, and 680 cm^−1^ belong to the stretching mode of Mo=O_1_, Mo-O_3_-Mo, and the Mo-O_2_-Mo (Figure 5B). The peaks appearing between 400 and 170 cm^−1^ refer to the different deformation vibrations of O=Mo=O and O-Mo-O, while those below 170 cm^−1^ are derived from the translation of rigid MoO_4_ chain. The monoclinic phase has a similar Raman spectrum, but at 500 cm^−1^ there is a band of the Mo-O valence vibration that is absent in the spectrum of the orthorhombic MoO_3_ and there are evident differences between o- and m-MoO_3_ in the ratio of the peaks, as well, mostly in the range of deformation vibrations. In the case of h-MoO_3_, the band at 1000 cm^−1^ and the triple peak located at 910–900–880 cm^−1^ are ascribed to the Mo=O valence vibrations, whereas the bands around 700, 500, 390, and 320 cm^−1^ refer to the O-Mo-O vibrations. The peaks below 300 cm^−1^ are attributed to the modes of the MoO_4_ tetrahedra chains [36,37,38,39].

The absorption properties of the phases were investigated by diffuse reflectance UV–Vis spectroscopy. In each spectrum, a distinct absorption edge can be observed, which is characteristic of semiconductors (Figure 5C). Every phase absorbs not only in the UV range, but also in the visible part of the spectrum. The hexagonal phase, due to its pale yellow color, absorbs only below 500 nm, while the blue colored orthorhombic phase has absorption also above 550 nm, and so does monoclinic MoO_3_. Based on the spectra, the band gaps were determined, which were 3.07 eV for h-MoO_3_, 3.33 eV for o-MoO_3_, and 3.02 eV for m-MoO_3_.

### 3.4. Gas Sensing

#### 3.4.1. Checking Crystalline Phases and Morphology at 500 °C

The gas sensing properties were tested between room temperature up to 500 °C. However, at lower temperatures the gas sensing signals were so small that they could not be evaluated properly. Therefore, the responses to various H_2_ gas concentrations were only recorded at 500 °C. Accordingly, the present study serves only as a short test about the gas sensing of hydrothermally prepared m-MO_3_, but later a more detailed study is needed with lower sensing temperatures and various test gases.

**Figure 6:**
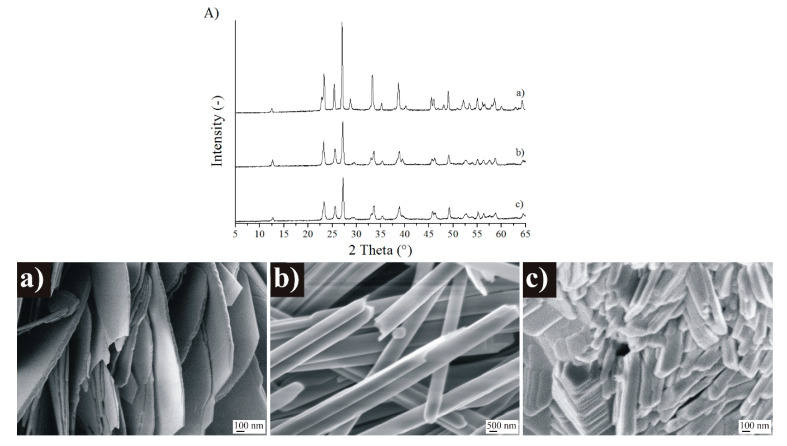
XRD patterns (**A**) and SEM images of different MoO_3_ phases after the 500 °C heat treatment., (**a**) MoO_3_-10, monoclinic phase (CrCl_3_, 240 °C, 6 h), (**b**) MoO_3_-8, orthorhombic phase (CTAB, 240 °C, 6 h), (**c**) MoO_3_-1, hexagonal phase (90 °C, 3 h).

Due to the relatively high operating temperature, before the gas sensing investigations, it was needed to check the possible phase and morphology transformations occurring at 500 °C, at the temperature of the gas sensing tests. Hence, all the MoO_3_ polymorphs had a 1 h heat treatment at 500 °C, and then they were studied by XRD and SEM (Figure 6).

Based on SEM and XRD results, we concluded that the heat treatment did not change the phase and morphology of o- and m-MoO_3_. However, the initially h-MoO_3_ phase transformed into o-MoO_3_, and the morphology changed, as well. The heated h-MoO_3_ (now o-MoO_3_) samples consisted of no hexagonal rods as before, but 10–20 nm wide, 200–300 nm long side-adhered plates, and irregular particles in various sizes (10–500 nm, Figure 6).

#### 3.4.2. Gas Sensing Tests

**Figure 7:**
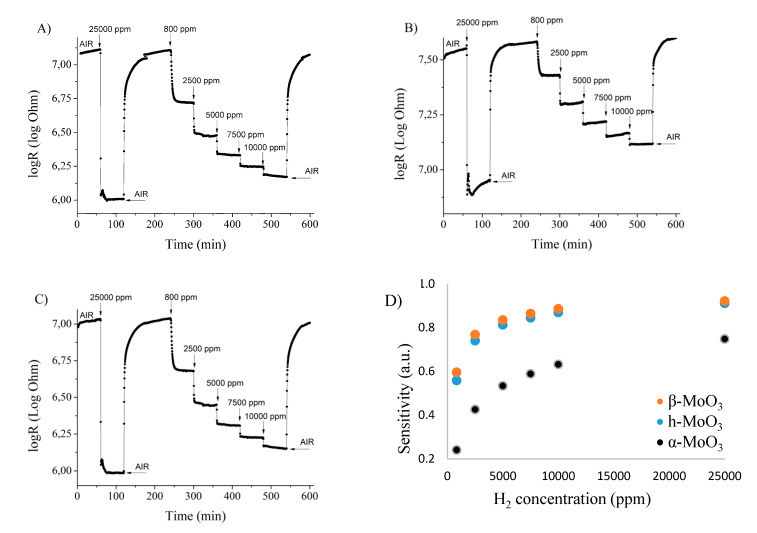
Results of the gas sensing test of the different MoO_3_ phases. (**A**) MoO_3_-10, monoclinic phase (CrCl_3_, 240 °C, 6 h); (**B**) MoO_3_-8, orthorhombic phase (CTAB, 240 °C, 6 h); (**C**) MoO_3_-1, hexagonal phase (90 °C, 3 h); (**D**) sensitivity of the MoO_3_ polymorphs.

During the tests the samples gave an n-type response, namely, their electrical resistance was reduced when exposed to hydrogen, which is typical for an n-type semiconductor reacting to a reducing gas [40]. The change in the logarithm of their electrical resistance as a function of time is presented in (Figure 7A–C). The sensitivity (*S*) of the sensors can be calculated using the following formula [41]: $S=\frac{\left|\Delta R\right|}{R_{base}}$, where ∆*R* is the change in electrical resistance due to the presence of the gas, while *R_base_* is the baseline electrical resistance of the material. As the baseline did not slip significantly, we selected the measured electrical resistance before the introduction of the 25,000 ppm H_2_ gas as R_base_. It can be observed that the sensitivity does not increase linearly with the increasing H_2_ concentration, and this might be related to the saturation of the gas sensing active sites (Figure 7D). Based on the gas sensing results, m- (β-) MoO_3_ is more sensitive to the H_2_ gas than o- (α-) MoO_3_ which has, however, a larger specific surface area. Probably the density of surface active site is different in their case, which can be explained by, e.g., different amounts and forms of pre-adsorbed oxygen (O^2−^, O^−^ or O_2_^−^) or the difference in surface OH groups similar to m- and h-WO_3_ [40,42]. The annealed h-MO_3_, which transformed into o-MoO_3_ at 500 °C has a similar sensitivity to the monoclinic polymorph. Probably the hexagonal-orthorhombic transformation caused an increase in the number of active sites or in the surface area, which might explain this.

The response time was defined as the time at which the electrical resistance reaches 90% of the maximum signal given to a certain gas concentration and the recovery time was defined as the time needed to return to 10% of the maximum signal after finishing the gas pulse. These values are given for each sample using 25,000 ppm H_2_ (Table 3). Based on the data, the response time of all samples is low, approx. 1 min. The samples with a similar sensitivity (monoclinic and transformed hexagonal) also have a similar recovery time, approx. 20 min. The recovery time was lower for the fiber-like o-MoO_3_ sample. This might be explained by its lower number of gas sensing active sites, which also explained the lower sensitivity of this sample.

## 4. Conclusions

In this study, we investigated the effect of various parameters on the hydrothermal preparation of MoO_3_, for the first time also at 240 °C, focusing on the changes in the crystalline phases and morphology of the products. We used different temperatures (90/210/240 °C) and durations (3/6 h) to investigate their role in our reaction system. At 240 °C, we also tested the effect of CTAB and CrCl_3_ additives, respectively. As a result, h-MoO_3_ formed at 90 °C independently on the duration and crystallized in the form of hexagonal rods. At 210 °C with 3 h reaction time, the product was a mixture of h- and o-MoO_3_, while with 6 h it was pure o-MoO_3_, alike at 240 °C, with nanofibrous morphology. With the use of the CTAB additive, we obtained pure o-MoO_3_ in the form of nanofibers, but with a radically reduced diameter due to the presence of the additive. However, when we applied CrCl_3_, it resulted in the metastable m-MoO_3_ phase at both reaction durations with nanosheet morphology. The gas sensing properties of the MoO_3_ samples against H_2_ were also tested. The m-MoO_3_ was found to be more sensitive than the o-MoO_3_.

## Figures and Tables

**Table 1:** Experimental conditions of the hydrothermal reaction between AHM and HNO_3_.

Sample	Additive	*T* (°C)	*t* (h)
MoO_3_-1	-	90	3
MoO_3_-2	-	90	6
MoO_3_-3	-	210	3
MoO_3_-4	-	210	6
MoO_3_-5	-	240	3
MoO_3_-6	-	240	6
MoO_3_-7	CTAB	240	3
MoO_3_-8	CTAB	240	6
MoO_3_-9	CrCl_3_	240	3
MoO_3_-10	CrCl_3_	240	6

**Table 2:** The specific surface area (SBET) and EDX results of the different MoO_3_ phases, MoO_3_-1: 90 °C, 3 h, MoO_3_-8: CTAB, 240 °C, 6 h, MoO_3_-11: CrCl_3_, 240 °C, 6 h.

Sample	SBET (m^2^/g)	EDX (Atom %)	
		Mo (Average)	O (Average)
MoO_3_-1, hexagonal	0.21	24.3	75.7
MoO_3_-8, orthorhombic	9.7	24.8	75.2
MoO_3_-10, monoclinic	2.9	25.7	74.3

**Table 3:** Response and return times of sensors using 25,000 ppm H_2_.

Sample	Response Time (min)	Recovery Time (min)
MoO_3_-10, monoclinic	1.2	22.5
MoO_3_-8, orthorhombic	1.2	10.1
MoO_3_-1, hexagonal	1.2	20.3
